# Draft Genome Sequence of Mortierella alpina Strain LL118, Isolated from an Aspen (Populus tremuloides) Leaf Litter Sample

**DOI:** 10.1128/MRA.00864-21

**Published:** 2021-11-24

**Authors:** Shu Yang, Boris A. Vinatzer

**Affiliations:** a School of Plant and Environmental Sciences, Virginia Tech, Blacksburg, Virginia, USA; University of Maryland School of Medicine

## Abstract

Mortierella alpina is a filamentous fungus commonly associated with soil and is one of very few fungal species known to include strains with ice nucleation activity. Here, we report the draft genome sequence of the ice nucleation-active *M. alpina* strain LL118, isolated from aspen leaf litter collected in Alberta, Canada.

## ANNOUNCEMENT

*Mortierella* species are filamentous fungi in the subphylum Mortierellomycotina (1). Mortierella alpina is the only *Mortierella* species that includes strains with ice nucleation activity (INA) (2, 3). Since INA plays a key role in atmospheric processes (4, 5), sequencing the genome of a *M. alpina* strain with INA is an important step toward understanding the possible contribution of *M. alpina* to atmospheric processes. This paper reports the genome assembly and transcriptome sequencing (RNA-seq)-based annotation of *M. alpina* strain LL118, a strain with INA that was isolated from an aspen (Populus tremuloides) leaf sample collected in Alberta, Canada (6).

Genomic DNA was extracted from mycelium grown in potato dextrose broth for 14 days at 28°C using the ZymoBIOMICS DNA miniprep kit (Zymo Research). Whole-genome sequencing was performed on an Illumina HiSeq 3000 platform at the Iowa State University DNA Facility with the Nextera DNA Flex library prep kit (Illumina), and the quality of reads was checked using FastQC v0.11.9 (7). All tools were run with default parameters unless otherwise specified. A total of 9.8 million 2 × 100-bp reads were generated. The reads were trimmed using Trimmomatic v0.39 (8) to remove adapters. *De novo* assembly was performed using SPAdes v3.15.2 (9) with -k 21,33,55,77 --careful --cov-cutoff auto. The completeness of the assembly was assessed using Benchmarking Universal Single-Copy Orthologs (BUSCO) v5.0.0 (10) with -m genome -l mucoromycota_odb10.

The genome assembly was found to have 54× coverage using BBMap v38.90 (11). The genome was determined to comprise 35,609,760 bp with a G+C content of 51.44%. The BUSCO assessment revealed the presence of 1,575 genes (97.6%) based on the lineage-specific profile library mucoromycota_odb10 (1,614 genes). The assembly statistics are shown in Table 1.

**TABLE 1:** Assembly summary and annotation features of *Mortierella alpina* strain LL118

Characteristic	Value^*a*^
Strain	F156N33
Assembly size (bp)	35,609,760
No. of contigs	2,222
Maximum contig length (bp)	504,467
Minimum contig length (bp)	200
Avg contig length (bp)	16,169
*N*_50_ contig length (bp)	96,689
GC content (%)	51.44
Assembly BUSCO coverage (%)	C: 97.6; F: 0.7; M: 1.7
Annotation BUSCO coverage (%)	C: 91.7; F: 2.1; M: 6.2
No. of predicted coding genes	8,852
Mean gene length (bp)	3,205

a BUSCO coverage: C, complete BUSCOs; F, fragmented BUSCOs; M, missing BUSCOs.

Total RNA was extracted from mycelium grown on potato dextrose agar for 30 days at 6°C, room temperature, and 28°C (three replicates for each temperature) using the RNeasy plant minikit (Qiagen). RNA-seq was performed on an Illumina Nova Seq 6000 platform at Novogene Corporation, Inc. (Sacramento, CA), using the TruSeq stranded mRNA library prep kit (Illumina), and the quality of reads was again checked using FastQC v0.11.9 (7). A total of 197.4 million 2 × 150-bp reads were generated. The RNA-seq reads were aligned to the genome assembly using STAR v2.7.8a (12) and assembled using Trinity v2.12.0 (13) and StringTie v2.1.5 (14).

The MAKER v3.01.03 pipeline (15) with SNAP v2013-02-16 (16) and AUGUSTUS v3.4.0 *ab initio* gene predictions (17) were employed for genome annotation using the assembled transcriptomes obtained above and the proteome of Linnemannia elongata (ID: UP000078512) from the UniProt database (18, 19). Functional annotations were performed using InterProScan v5.46-81.0 (20) and BLASTP from BLAST v2.10.0+ (21). The completeness of the genome annotation was assessed using BUSCO v5.0.0 (10) with -m proteins -l mucoromycota_odb10.

The BUSCO assessment revealed the presence of 1,480 complete genes (91.7%) and 34 fragmented genes (2.1%) (Table 1), indicating a good-quality genome annotation. A total of 8,852 protein-coding genes were predicted. Of the predicted proteins, 7,288 (82.3%) received a functional annotation.

### Data availability.

The draft genome sequence has been deposited in DDBJ/ENA/GenBank under accession number JAIFTL000000000.1, BioSample accession number SAMN20056918, and BioProject accession number PRJNA743604. The raw DNA sequences have been deposited in the Sequence Read Archive (SRA) under accession number SRR15443011, and the raw RNA sequences can be found under accession numbers SRR16290171, SRR16290170, and SRR16290169.
